# Interventions to improve cancer survivorship among Indigenous Peoples and communities: a systematic review with a narrative synthesis

**DOI:** 10.1007/s00520-021-06216-7

**Published:** 2021-05-24

**Authors:** Wendy Gifford, Margo Rowan, Peggy Dick, Shokoufeh Modanloo, Maggie Benoit, Zeina Al Awar, Liquaa Wazni, Viviane Grandpierre, Roanne Thomas, Lindsey Sikora, Ian D. Graham

**Affiliations:** 1 School of Nursing, Faculty of Health Sciences, University of Ottawa, Ottawa, Ontario Canada; 2 Rowan Research and Evaluation, Ottawa, Ontario Canada; 3 Algonquins of Pikwakanagan Health Services and Family Health Team, Pikwakanagan, Ontario Canada; 4 School of Rehabilitation Sciences, Faculty of Health Sciences, University of Ottawa, Ottawa, Ontario Canada; 5 Health Sciences Library, University of Ottawa, Ottawa, Ontario Canada; 6 School of Epidemiology and Public Health, Faculty of Medicine, University of Ottawa, Ottawa, Ontario Canada; 7 Ottawa Hospital Research Institute (OHRI), University of Ottawa, Ottawa, Ontario Canada

**Keywords:** Indigenous Peoples, Cancer survivorship, Healthcare interventions, Systematic review

## Abstract

**Purpose:**

The purpose of this systematic review is to synthesize the evidence on the types of interventions that have been utilized by Indigenous Peoples living with cancer, and report on their relevance to Indigenous communities and how they align with holistic wellness.

**Methods:**

A systematic review with narrative synthesis was conducted.

**Results:**

The search yielded 7995 unique records; 27 studies evaluating 20 interventions were included. The majority of studies were conducted in USA, with five in Australia and one in Peru. Study designs were cross-sectional (*n*=13); qualitative (*n*=5); mixed methods (*n*=4); experimental (*n*=3); and quasi-experimental (*n*=2). Relevance to participating Indigenous communities was rated moderate to low. Interventions were diverse in aims, ingredients, and outcomes. Aims involved (1) supporting the healthcare journey, (2) increasing knowledge, (3) providing psychosocial support, and (4) promoting dialogue about cancer. The main ingredients of the interventions were community meetings, patient navigation, arts, and printed/online/audio materials. Participants were predominately female. Eighty-nine percent of studies showed positive influences on the outcomes evaluated. No studies addressed all four dimensions of holistic wellness (physical, mental, social, and spiritual) that are central to Indigenous health in many communities.

**Conclusion:**

Studies we found represented a small number of Indigenous Nations and Peoples and did not meet relevance standards in their reporting of engagement with Indigenous communities. To improve the cancer survivorship journey, we need interventions that are relevant, culturally safe and effective, and honoring the diverse conceptualizations of health and wellness among Indigenous Peoples around the world.

**Supplementary Information:**

The online version contains supplementary material available at 10.1007/s00520-021-06216-7.

## Introduction

Worldwide, Indigenous Peoples^1^ have a higher cancer burden than non-Indigenous counterparts [1–8]. One report indicated that cancer incidence and mortality rates were higher in First Nations people in Canada for lung, colorectal, and kidney cancers as compared to non-First Nations people [2], while a comparison of 5-year survival and mortality rates for 15 cancers in a cohort population across Canada found that First Nations adults had poorer survival from all cancers except multiple myeloma, reaching over 20% lower for cervical and ovarian cancers and 10–15% lower for colorectal, breast, non-Hodgkin lymphoma, and leukemia [9]. In the province of Manitoba, First Nations people diagnosed with cancer were significantly younger with significantly higher mortality rates, despite similar incidence rates after adjusting for age, sex, income, and area of residence [8]. The underlying causes of these disparities are wide ranging and complex, and include lower rates of screening and late-stage diagnoses [10, 11]. However, more pervasive reasons stem from colonial legacies that have created poverty, social exclusion, and systemic racism in mainstream healthcare [1, 2, 11–14].

The unique cancer burdens faced by Indigenous Peoples have largely been attributed to the impacts of colonization and the subsequent lack of culturally safe healthcare services and supports [1, 2, 12, 13]. Historical trauma has profoundly impacted Indigenous peoples’ trust and engagement in Western healthcare systems [1, 15, 16]. Approaches to survivorship that are tailored to specific needs of survivors have been shown to decrease cancer burden, increase survival rates, and enhance well-being [1, 17]. Despite potential benefits, Indigenous Peoples do not typically seek survivorship supports and Indigenous values, practices, and distinct needs are not typically reflected in mainstream health care or cancer survivorship interventions [1, 15, 17–20]. While the term “cancer survivorship” can vary, we recognize it as living with, through, and beyond a diagnosis of cancer [21]. Many Indigenous people have described feeling unsafe and fearing stigmatization with cancer survivorship interventions [17]. In voicing their survivorship experiences, Indigenous people have described a failure of healthcare services to accommodate their distinct ethnic, cultural, and socio-historical needs [17, 18].

What might culturally safe cancer survivorship programs and supports look like for Indigenous Peoples? The Health Council of Canada recommends the provision of culturally safe care that respects traditional, holistic approaches to wellness and healing [18]. The incorporation of traditional, holistic approaches that include spirituality, traditions, and family has been found to be important to Indigenous cancer survivors [17, 22, 23]. Indigenous people are in the best position to guide their path to health and wellness [24], and strengths-based, community engaged, decolonizing approaches that recognize and honor Indigenous knowledge are needed [25].

Grounding cancer survivorship interventions within an understanding of Indigenous wellness is also important [2]. Indigenous health and wellness are often understood to be a balance of one’s physical, spiritual, emotional, and mental being. While this may be expressed differently in different Indigenous nations, it is closely tied to the four dimensions of the Medicine Wheel for First Nations Peoples in North America [26, 27].

Setting a course to move cancer survivorship programs forward calls for a comprehensive exploration of past initiatives. Cancer survivorship interventions that have been used by Indigenous Peoples have not been systematically described, particularly their relevance to Indigenous communities and Indigenous wellness. As part of a larger study to improve healthcare deliver with Indigenous Peoples in Canada [28], the purpose of this systematic review was to synthesize the research evidence on cancer survivorship strategies that have been used by Indigenous Peoples. The research objectives are to:

1. Identify methodological approaches that have been used in cancer survivorship research,
2. Describe components of cancer survivorship interventions and the reported evidence on their relevance to Indigenous communities,
3. Examine outcomes of the interventions and their positioning to holistic wellness.

## Methods

We considered quantitative and qualitative evidence following a multi-stage methodological approach that involved searching the literature, screening articles for inclusion and exclusion criteria, extracting data, assessing articles for methodological quality and relevance to Indigenous communities, and synthesizing study findings to produce a narrative summary of results [29].

### Search strategy

We created the search strategy with a health sciences librarian. Seven electronic databases were searched from their date of inception to March 6, 2018, and then were updated on August 20, 2020: Medline (Ovid), Embase (Ovid), Cochrane’s Central Registry for Randomized Controlled Trials - CENTRAL (Ovid), PsycINFO (Ovid), CINAHL (EBSCOHost), and PubMed. No modifications were made to the search strategy during the update. We included keywords and subject heading applicable to each database for concepts related to Indigenous Populations, cancer survivorship, and interventions (See Additional files 1 and 2). Supplemental searching involved examining reference list of eligible articles and systematic reviews identified in the search.

#### Types of studies

We included articles with quantitative, qualitative; and mixed-methods designs. To be included, studies had to (1) involve Indigenous cancer survivors or caregivers, (2) execute a psychosocial cancer survivorship intervention, (3) report on patient outcomes qualitatively and/or quantitatively, and (4) collect primary data. There were no language exclusion criteria, and no restrictions on publication date.

Systematic reviews, commentaries, editorials, and theses were excluded. Studies were also excluded if the interventions involved treatments such as pharmacological, surgical, radiation, biological, or stem cells as this review was concerned with survivorship supports and not treatments. Interventions that focused on pre-diagnosis screening were also excluded.

### Selection of studies

All titles and abstracts identified in the database search were independently screened for eligibility in Covidence systematic review software [30] by four reviewers. Full copies of articles identified as potentially eligible, or with insufficient information to decide, were retrieved and independently assessed for inclusion criteria by two reviewers. Disagreements were resolved through discussions and adjudication with the lead author (WG).

### Data extraction

We designed a data extraction form based on the review objectives and iteratively refined it after pilot testing with two articles from each study design (*n*=6) to ensure the data extracted reflected the aims of the review. One reviewer extracted data from all included articles into an Excel spreadsheet and two reviewers verified it for accuracy. Data were extracted on study characteristics, including outcomes and impacts, and any information about collaborating with Indigenous communities to conduct the study. Details about each intervention were extracted into categories of the *AIMD* framework that describe (1) Aims (what the intervention is intended to achieve and for whom); (2) Ingredients (what comprises the intervention); (3) Mechanisms (how the intervention is proposed to work); and (4) Delivery (how the intervention was delivered) [31]. The *AIMD* framework was developed to enhance understandings of how interventions work to inform healthcare practices and policies [31].

### Assessment of relevance to Indigenous communities

Two reviewers (SM, LW) independently assessed each study’s relevance to Indigenous communities and methodological quality. Discrepancies were resolved and assessments confirmed through discussions and adjudication with two authors (WG, MR) who provided the final assessment ratings. We used a tool inspired by Smylie et al. to assess the evidence of the interventions’ relevancy to participating Indigenous communities and members [32, 33]. The assessment examined whether there was evidence in the published papers of Indigenous participants’ engagement in the research through four categories of relevance: (1) alignment of study designs and measures to the community/participants’ values, beliefs, and knowledge systems; (2) alignment to local priorities; (3) relevance of underlying intervention to participating communities/participants; and (4) whether the study protocol was vetted by local community members [32, 33]. We measured evidence of relevance on a zero-to-eight scale by summing scores of community participation in any stage of the study, including development and implementation. Each of the four categories was scored as follows: 0=not reported, 1=partial evidence; 2=explicit evidence. A composite score was created for each intervention by totalling the four subcomponent scores where 0=none, 1–3=weak, 4–6=moderate, and 7–8=strong evidence of the interventions relevance to participating communities.

### Assessment of methodological quality

We used three tools to assess methodological quality of included studies according to study design: (1) *McMaster Critical Review tools* for quantitative research [34]; (2) *McMaster Critical Review tools* for qualitative research [35]; and (3) *Mixed Methods Appraisal tool* (MMAT) for mixed-methods studies [36, 37]. We adapted a scoring system based on previously published systematic reviews [38, 39]. We divided the number obtained in the quality rating for each study by the total number of possible points, to obtain a total quality rating between 0 and 1. Studies were then categorized as weak (0–.25), weak-moderate (.26–.50), moderate (.51–.75), or strong (.76–1.0).

We conducted methodological quality and relevance assessments for each study that evaluated an intervention and aggregated data (when required) for all studies that evaluated the same intervention. For example, if one intervention was evaluated in three studies, we assessed the methodological quality and relevence to Indigenous communities or participants from the pooled data of all the studies that evaluated the same intervention. (See Table 1 for a summary of tools).

**Table 1:** Summary of tools used to assess relevence to Indigenous communities and methodological quality of studies

Name of tool	Purpose of tool	Key categories	Scoring system
Evidence of relevance to Indigenous communities (36, 37)	To assess transparency in reporting of the interventions’ relevancy to participating Indigenous communities and members	•Alignment of study design and measures to community or participants’ values, beliefs, and knowledge systems •Alignment to local priorities •Relevance of intervention to participating communities or participant •Vetting of study protocol by local community members	Each item was rated as: 0=not reported, 1=partial evidence; 2=explicit evidence. Ratings were totalled to create a composite score where: 0=none, 1–3=weak, 4–6=moderate, 7–8=strong evidence of the interventions’ relevancy to participating Indigenous communities and members
McMaster Critical Review tools: Quantitative (38)	To assess methodological quality of quantitative studies	•Selection bias •Study design •Confounders •Blinding •Data collection methods •Withdrawals and dropouts •Intervention integrity •Analysis appropriation	Each item was rated as 0 (not present) or 1 (present) and the number obtained divided by the total number of possible points to obtain a score of 0–1. Studies were categorized as: weak (0–.25), weak-moderate (.26–.50), moderate (.51–.75), strong (.76–1.0).
McMaster Critical Review tools: Qualitative (39)	To assess methodological quality of qualitative studies	•Study purpose •Relevance of literature •Study design •Sampling •Data collection clarity •Data collection procedural rigor •Analytical rigor •Auditability •Theoretical connections •Overall rigor •Conclusions and implication	Each item was rated as 0 (not present) or 1 (present) and the number obtained divided by the total number of possible points to obtain a score of 0–1. Studies were categorized as: weak (0–.25), weak-moderate (.26–.50), moderate (.51–.75), strong (.76–1.0).
Mixed Methods Appraisal tool (MMAT) (40, 41)	To assess methodological quality of mixed methods studies	•Clarity of research questions •Qualitative approaches •Quantitative randomization/blinding/confounders •Quantitative sampling/measures/analysis •Mixed-methods approaches	Each item was rated as 0 (not present) or 1 (present) and the number obtained divided by the total number of possible points to obtain a score of 0–1. Studies were categorized as: weak (0–.25), weak-moderate (.26–.50), moderate (.51–.75), strong (.76–1.0).

*RCTs* randomized control trials

### Data synthesis

We conducted a narrative synthesis [40] to produce a summary of studies. We tabulated characteristics of included studies in Excel and descriptively synthesized data on interventions and outcomes. To synthesize components of the interventions, we systematically coded data into the following categories based on the *AIMD* framework: Aims, Target Group, Ingredients, and Delivery. We then developed themes for each of the categories as they emerged from the studies. Themes were based on the primary author’s descriptions whenever possible. For example, when an author reported that the purpose of the intervention was to “*better support Indigenous Australians through their cancer journey*,” we identified the theme as “*support and improve the healthcare journey*.”

As methodologies, interventions, and outcomes were vastly heterogeneous, we categorized outcomes into descriptive themes as they related to the outcomes that were evaluated. Indigenous team members (ND, MB) brought an Indigenous perspective to the thematic analysis of outcomes. We first categorized outcomes as follows: (1) *holistic wellness outcomes* and (2) *responses towards the intervention. Holistic wellness outcomes* corresponded to conceptualizations of Indigenous wellness and encompassed findings related to physical, mental, emotional, or spiritual health. The physical domain involves taking care of one’s body; the mental involves rational thought; the emotional encompasses relationships and being connected to family and community; and the spiritual involves beliefs, values, and identity [26, 27, 41]. *Responses towards the intervention* encompassed aspects of the interventions that could influence wellness, such as perceptions of cultural safety, cultural appropriateness, acceptability, or satisfaction. We then inductively created subcategories for each outcome category as they emerged from the data, using an iterative and consultative process among the research team that involved data display, re-categorization, and confirmation.

## Results

**Fig. 1:**
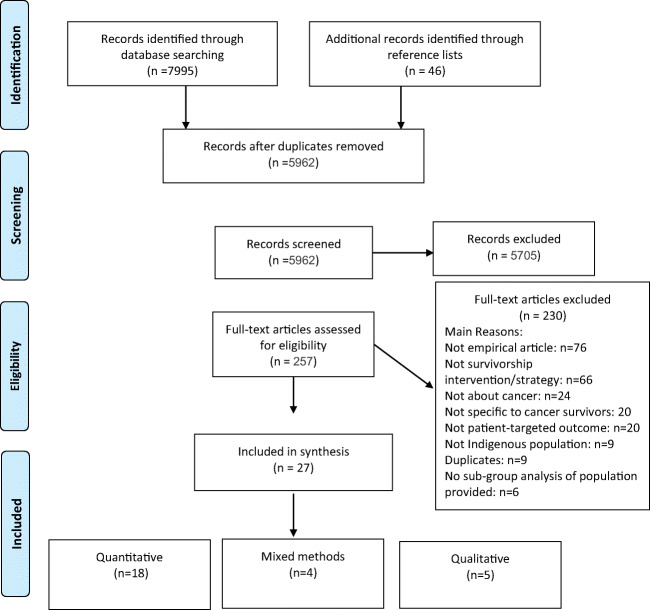
PRISMA flow diagram

The search yielded 7995 unique records after duplicate removal, with *257* identified as potentially relevant. Twenty-seven studies evaluating 20 interventions met the inclusion criteria and were included in this review. Of the potentially relevant articles that were not included, the top reasons were not empirical involving primary data (*n*=76, 33%), no intervention (*n*=66, 28%), or not about cancer (*n*=24, 10%) (Fig. 1 PRISMA diagram).

### Characteristics of included studies

Twenty interventions were evaluated in 27 studies. Characteristics of included studies are described in Table 2. Four interventions were evaluated in multiple studies, i.e., (1) *Native Navigators and Cancer Continuum* (NNACC) program [42–44]; (2) *Walking Forward* program [45–48]; (3) *Family ‘ohana intervention* [23, 49], and (4) *Aboriginal women’s cancer support network* [50, 51]. The remainder of the interventions were evaluated in a single study. Sixty-seven percent of the studies were quantitative (*n*=18) [23, 42–49, 52–58], 19% qualitative (*n*=5) [50, 51, 59, 60], and 15% mixed methods (*n*=4) [61–64].

**Table 2:** Characteristics of included studies

Intervention/program: name and description	Evidence of relevance to Indigenous communities*	Study(s) First author, year	Country and setting	Sample size, participants, ethnicity, age, sex	Study design** Collection methods	Quality assessment rating*** (Component weakness)
**The Cancer Healing Messages flipchart and patient flyer** •Designed according to principles of best practice for developing acceptable and useful resources for Aboriginal communities.	Moderate • Intercultural approach with Indigenous healthcare service providers. • Community-driven culturally relevant intervention.	Bierbaum (2017)	Australia, Organizations that deliver health services to Aboriginal people	•*N* = 18 • Health care providers • Australian Aboriginal and Torres Strait Islander •Age= unspecified •67% female, 33% male	Non-experimental •Survey	Weak-moderate •Validity and reliability of data collection tools not reported. •Numbers and reasons for dropouts not indicated. •Consistency of intervention not reported.
**Native Navigators and the Cancer Continuum (NNACC)** •Refines, expands, and adapts various navigator/community education programs to address Native American communities’ and members’ needs throughout continuum of cancer care: • Educational workshops—24 h of content in a series of 3–6 workshops to encourage healthy behaviors, increase participants’ knowledge, and address barriers to screening or healthy behaviors. • Family Fun Events—held in conjunction with each workshop series, to promote the workshops, collect data, assess knowledge retention, and disseminate workshop findings.	Moderate • Navigators and collaborators from community. • Local navigators informed workshop topics. • Intervention theory adaptable to local community needs.	Burhansstipanov (2012)	USA, Community	•*N*=900 •Survivors •Native American •18+ yrs. •75% female.	Non-experimental •Survey (pre/post)	Weak-moderate •Validity and reliability of data collection tools not reported. •Numbers and reasons for dropouts or withdrawals not indicated. •Consistency of intervention not reported.
		Burhansstipanov (2014)	USA, Community	•*N*=1964 •Survivors •Native American •18–95 yrs. •70% female.	Non-experimental •Survey (pre/post)	
		Krebs (2013)	USA, Community	•Survivors and Healthcare Providers • *N*, age, and sex unspecified.	Non-experimental •Survey (pre/post)	
•**Native American Navigator Program** • Navigators called *Native Sisters* assist clients identify mammography facilities, obtain transportation and childcare, and translate information. • Education Brochure of risk factors, e.g., age of menopause, family history of breast cancer.	Moderate • Materials reviewed by community and cultural components added. • Participants supported by local Indigenous navigators during care	Dignan (2005)	USA, Community	•*N*=157 •Survivors •Native American •Average=54 yrs. •100% female.	Experimental (RCT) •Survey (pre/post)	Weak-moderate •Confounding differences between groups not reported. •Validity and reliability of data collection tools not reported. •Consistency of intervention not reported. •Intention-to-treat not reported for analyzing results (where all participants randomized are included in analysis, and analyzed according to originally assigned group, regardless of what treatment, if any, they received).
**American Indian navigation program** •Help AI patients negotiate the Indian Health Service (IHS) system	Weak • Community relevant intervention.	Dockery (2018)	USA, Hospital	•*N* = 55 •Survivors • Native American •Median age= 45 yrs. •100% female	Non-experimental •Medical records—retrospective review	Weak-moderate •Validity and reliability of data collection tools not reported. •Consistency of intervention not reported.
**Telehealth support group service** •Monthly 2-h group counseling and education sessions (*n*=12) • Content included group counseling, education modules, and presentations by content experts.	Weak • Local coordinators facilitated groups. • Educational contents developed with local community.	Doorenbos (2010)	USA, Community	•*N* = 32 •Survivors and caregivers •Average=53 yrs. • Native American and Alaska Natives •100% female	Non-experimental •Survey (post)	Moderate •Validity and reliability of data collection tools not reported. •Consistency of intervention not reported.
**Video intervention** •For rural Peruvian women with cervical neoplasia before loop excisional procedures. •Culturally sensitive video discusses lower genital tract anatomy; cervical neoplasia; and the indications, preparation, procedure, complications, and postoperative information on loop excision surgery.	Weak • Culturally sensitive video made available in native language.	Ferris (2015)	Peru, Medical Clinic	•*N*= 81 •Survivors •Peruvian •18+ yrs. •100% female	Quasi-experimental •Survey (pre/post)	Weak-moderate •Validity and reliability of data collection tools not reported. •Numbers and reasons for dropouts or withdrawals not indicated. •Consistency of intervention not reported.
***Walking Forward*** **patient navigator program -*To’katakiya zanniyan omani pi ye/yo*** • Navigators (hospital-based and community) assist: • Navigating therapy • Obtaining medications • Insurance issues • Communication with medical providers •Travel logistics •Psycho-social support. •Community research representatives provide cancer education, network with local health resources, collect data, and serve as liaisons between cancer center, patient navigators, and patients or tribal governments.	Moderate • Community reps involved in delivering intervention, collecting data, developing liaisons. • Patient materials translated into native language. • Tribal leadership and Indigenous health organization consulted from onset.	Guadagnolo & Boylan (2011)	USA, Hospital	•*N*=402 •Survivors • Native American • Unspecified •55% female	Non-experimental •Medical records	Moderate •Outcome assessors not blinded. •Numbers and reasons for dropouts or withdrawals not indicated. •Consistency of intervention not reported.
		Guadagnolo & Cina (2011)	USA, Hospital	•*N* = 52 •Survivors • Native American •18+ yrs. •40% female	Non-experimental •Survey (pre/post)	
		Petereit (2008)	USA, Community	•*N* = 213 •Survivors • Native American • Unspecified • Unspecified	Non-experimental •Survey (pre/post)	
		Petereit (2011)	USA, Community	• *N*= 332 • Survivors and caregivers • Native American • Unspecified • Unspecified	Non-experimental •Survey (pre/post)	
**Cancer 101: An Educational Program for Native Settings** Content includes: •Cancer concerns •What is cancer? •Screening and early detection •Diagnosis and staging •Risks and risk reduction •Basics of treatment •Support for patients and caregivers.	Moderate • Educational materials tailored to community needs. • Tribal community liaisons involved in recruitment, tailoring and dissemination of materials to community.	Hill (2010)	USA, Community	•*N* =70 •Healthcare Providers • Native American and Alaska Native •Unspecified. •89% female	Non-experimental •Survey (pre/post)	Weak-moderate •Validity and reliability of data collection tools not reported. •Consistency of intervention not reported.
**Symptom management toolkit** • Symptom management booklet/resource guide: •Cancer etiology •Diagnosis, treatment, and follow-up •Cancer symptoms management •Strategies and tips for pain, body changes, and changing activity levels •Recommendations on how to communicate with providers, family members, and others. •Self-management video: •Cancer facts /myths •Coping with cancer •What to expect after diagnosis and treatment •Support systems •Strategies/tips for pain relief, fatigue, depression, function • Spirituality and balance	Moderate • Educational materials tailored to community needs. • Tribal community liaisons involved in recruitment, tailoring and dissemination of materials to community.	Hodge (2016)	USA, Community	•*N*= 184 •Survivors and caregivers • Native American •18+ yrs. •70% female	Quasi-experimental •Survey (pre/post)	Weak-moderate •Validity and reliability of data collection tools not reported. •Numbers and reasons for dropouts or withdrawals were not indicated. •Consistency of intervention not reported.
**Family ‘Ohana educational** •To build family capacity by improving knowledge and skills of breast cancer survivors and their family. •Emphasis on later stage of recovery care. • 4, 2-h educational sessions over a 4-month period, including materials and training to access information on cancer and communicate with health care providers.	Moderate • Intervention materials culturally tailored. • Local community food supported “feeding the spirit”. • Stories used with cultural discussion groups.	Mokuau (2008)	USA, Community	•*N* = 28 •Survivors and caregivers •Native Hawaiian •Average=55 yrs. •100% female	Experimental (RCT) •Survey (pre/post)	Moderate •Confounding differences between groups not reported. •Consistency of intervention not reported.
		Mokuau (2012)	USA, Community	•*N*= 58 •Survivors and caregivers •Native Hawaiian •Unspecified •100% female	Experimental (RCT) •Survey (pre/post)	
**Telemedicine-based counseling program** • Interactive audio and video telemedicine program for high-risk patients with breast cancer. • Included telehealth coordinators and navigators	Weak • Tribal health consortium involve in telemedicine platform.	Pruthi (2013)	USA, Medical clinic	•*N*= 58 •Survivors •Alaska Native • Unspecified •100% female	Non-experimental •Survey (cross-sectional)	Weak •Confounding differences between groups not reported. •Validity and reliability of data collection tools not reported. •Numbers and reasons for dropouts or withdrawals not indicated. •Consistency of intervention not reported.
**Townsville teleoncology clinic** • Telemedicine for rural cancer care involving videoconferencing consultation sessions with patients.	None • Not identified.	Sabesan (2012)	Australia, Hospital	•*N*=158 •Survivors • Australian Aboriginal •Unspecified •52% female.	Non-experimental •Electronic databases •Medical records	Weak •Confounding differences between groups not reported. •Validity and reliability of data collection tools not reported. •Numbers and reasons for dropouts or withdrawals not indicated. •Consistency of intervention not reported.
**Aboriginal women’s cancer support network** •Facilitated access to services •Fostered social interaction •Provided culturally safe space •Built working relationships with services and agencies.	Weak • Intercultural approach with Indigenous healthcare service providers.	Cuesta-Briand (2015)	Australia, Community	•*N* = 24 • Healthcare Providers •Australian Aboriginal •Unspecified •92% female	Unspecified qualitative •Interviews	Strong •No theoretical perspective identified. •Role of researcher and relationship with participants not described. •Process of development of decision trail not identified.
		Cuesta-Briand (2016)	Australia, Community	•*N* = 24 • Healthcare Providers • Australian Aboriginal •Unspecified •92% female		
**Cancer symptom self-management toolkit** • Self-directed guidebook • Resource directory • Motivational video.	Moderate • Cultural constructs explored for toolkit. • Community representatives involved in framework development and validation • Video pilot tested for cultural appropriateness	Hodge (2012)	USA, Community	•*N* = 132 •Survivors and their family • Native American (Southwest) •18+ yrs. •72% females.	Grounded theory •Focus groups	Strong •No theoretical perspective identified. •Role of researcher and relationship with participants not described. •Purposeful sampling selection not described.
**Cancer care team (CCT):** •Improving cancer care for Australian Aboriginal patients • The cancer care team consisted of an Australian Aboriginal health worker, counsellor, and enrolled nurse employed for 2 days a week, supported by a GP. •Included follow-up of abnormal test results, Support at first diagnosis, Yarning circle, Palliative care, Carer support, Prevention programs.	Moderate • Stories used with cultural discussion groups • Intercultural approach with Indigenous healthcare service providers. • Community-driven culturally relevant intervention.	Ivers (2019)	Australia, An Australian Aboriginal community-controlled health service in New South Wales.	•*N* = 8 •Health care provider, clients, and stakeholder • Australian Aboriginal •Age= 54 to 81 yrs. •90% female, 10% male	Ground theory •Semi-structured interviews	Moderate •Purposeful sampling selection not described. •Role of researcher and relationship with participants not described. • Not well addressed theoretical perspective
**Reader's theater** • Cancer education where two or more people read aloud while listening to a scripted conversation. •Readers are arranged among listeners to create a conversational, inclusive experience.	Moderate • Oral tradition used as a way of teaching. • Used culturally respectful, holistic ways of inquiry.	Cueva (2010)	USA, Community	•*N*= 24 •Survivors and caregivers •Alaska Native (Athabascan, Tlingit, Inupiat, Yup’ik, Aleut, Chippewa) •Average=45 yrs. •92% female.	Organic inquiry design •Interviews •Journaling •Field notes •Written reflections and discussions.	Weak-moderate •Purposeful sampling selection not described. •Role of researcher and relationship with participants not described. •Sufficient description of participants and site not described. •Process of inductive analysis and development of decision trail not described. •4 elements of study rigor/trustworthiness not described (Credibility, Transferability, Dependability, Confirmability).
**Reader's theatre** •45-min play to promote cancer education as a collaborative effort, incorporating stories shared by people throughout Alaska.	Weak • Collaborated with community to make play • Storytelling used to understand community’s way of knowing.	Cueva (2005)	USA, Community	•*N*=401 •Survivors •Alaska natives •40+ yrs. • 85% female.	Mixed-methods triangulation •Survey (post) •Discussions.	Weak-moderate •Qualitative and quantitative aspects not effectively integrated to answer research question. •The consistency of intervention not reported.
**Enhancing Cancer Pain Control among Indians (ECPCAI)** • Culturally sensitive brochures for patients • Guidelines for providers. • Continuing Medical Education (CME) for healthcare providers.	Moderate • Tribal groups, Elders, and traditional healers involved in creating culturally appropriate materials. • Instruments tailored in consultation with tribal group. • Cultural components discussed to ensure relevance and sensitivity of materials.	Elliott (1999)	USA, Community	•*N*=128 •Healthcare Providers and Elders • Native American (Anishinabe) •Unspecified •Unspecified	Mixed Methods exploratory •Focus groups •Interviews •Survey.	Moderate •Qualitative and quantitative aspects not adequately integrated. •Outcome assessors not blinded to intervention. •Cofounders not accounted for analysis.
**Culturally specific educational video:** ***Breast Cancer: It Can Be Healed*** • Narrated in Navajo language with English subtitles • Provides information on breast cancer treatment options, • Produced by a Navajo breast cancer survivor and oncology nurse.	Weak • Video translated into native language.	Sanderson (2010)	USA, Community	•*N* = 40 •Survivors and healthcare providers • Native American (Navajo) •18+ yrs. •100% female.	Mixed-methods triangulation •Interviews •Survey (post).	Weak-moderate •Numbers and reasons for dropouts or withdrawal were not reported. •Cofounders not accounted for analysis. •Qualitative and quantitative aspects not adequately integrated.
**Healing Pathways** •Art therapy project that brings American Indian cancer survivors and their family members together. •3 workshops promote stress reduction behaviors.	Moderate • Community-driven culturally relevant intervention. • Emergent design allowed tribal members to revise workshops for better alignment with survivor needs.	Warson (2012)	USA, Community	•*N* = 46 •Survivors • Native American •Unspecified •100% female.	Mixed-methods triangulation • Survey (pre/post) •Thematic analysis of art pieces • Interviews.	Moderate •Qualitative and quantitative aspects not effectively integrated to answer research question. •Outcome assessors not blinded to intervention.

*Evidence of relevance to Indigenous communities: 0=none, 1–3=weak, 4–6=moderate, 7–8=strong

**Design: label identifies the most prominent study design. Non-experimental designs include cross-sectional or pre-experimental studies in which there is no randomization and no control group. Quasi-experimental designs lack full control or randomization for example of participants, location, and timing of the intervention. Experimental designs use randomization and control, for example randomized control trial (RCT). Qualitative designs: label from cited paper where specified. Mixed-methods designs: label assigned according to mixed methods design types identified by Creswell and Clark (2007).

***Quality assessment rating: 0–.25= weak, .26–.50=weak-moderate, .51–.75= moderate, .76–1.0=strong

Of the 18 quantitative studies, three (*n*=17%) used an experimental design (RCTs) [23, 49, 56] and the remainder used quasi-experimental or non-experimental designs. All quantitative studies used surveys for data collection except for two [45, 53], which used medical records and/or electronic databases. Data in the mixed-methods studies (*n*=4) involved surveys, interviews, or discussion groups, while the qualitative studies (*n*=5) employed interviews, focus groups, and journaling.

Twenty-one (77%) of the included studies were conducted in the USA, with five from Australia (18%), and one from Peru (4%). Studies had diverse healthcare settings, the majority set in the community (*n*=21, 77%), four in hospitals, and two in medical clinics.

The total number of participants in each study ranged from 8 to 900. Participants’ Indigenous ethnicity included Native American (*n*=13; 50%), Alaskan Native (*n*=3; 12%), a combination of Native American and Alaskan Native (*n*=2; 8%), Native Hawaiian (*n*=2; 8%), Australian Aboriginal (*n*=5; 19%), Peruvian (*n*=1; 4%), and not specified (*n*=1, 4%). Twenty-six percent of studies (*n*=7) included family members or caregivers. Only one study reported the majority of participants as male [46], and four studies (15%) did not report the sex of participants [44, 47, 48, 63]. The earliest study was published in 1999 [63] with 81% published over the last 10 years (*n*=22).

### Relevance to communities

Ratings of relevance to participating Indigenous communities were as follows: eight studies rated as weak [51, 52, 57, 58, 62, 64–66], 18 studies as moderate [23, 42–49, 54–56, 59–61, 63, 67, 68], and one study provided no evidence of relevance [53]. Overall, ratings were highest for studies keeping with local community beliefs, values, and local priorities as evident by community members’ participation in designing, tailoring, or delivering the interventions and collecting study data. Having study protocols vetted by community members was the least reported relevance criterion.

### Methodological quality assessment

Quality assessments for the three experimental studies were moderate [23, 49] and weak-moderate [69]; quasi-experimental studies were weak-moderate [55, 58]; and non-experimental studies were moderate (*n*=5) [45–48, 57], weak-moderate (*n*=6) [42, 43, 54, 65, 68, 70], and weak (*n*=2) [52, 53]. For qualitative studies (*n*=5), three rated strong [51, 60, 66], one moderate [67], and one weak-moderate [59]. Of the mixed-methods studies (*n*=4), two rated moderate [61, 63] and two rated weak-moderate [62, 64]. Discrepancies in methodological quality assessment varied according to study design, with issues in quantitative studies predominately related to the validity and reliability of data collection tools, intervention integrity, numbers and reasons for dropouts or withdrawals, and confounding differences between groups. In the qualitative studies, quality assessment concerns involved inadequate reporting of theoretical perspectives, role of researchers, and relationship with participants. Clarity on how qualitative and quantitative aspects were integrated in mixed-methods studies had the weakest ratings (see Table 2).

### Interventions

The interventions were diverse in aims, ingredients, and outcomes studies. The overarching aims of the interventions were (1) to support and improve the healthcare journey (*n*=19 studies); (2) increase knowledge (*n*=8 studies); (3) provide psychosocial support (*n*=6 studies); and (4) promote dialogue about cancer (*n*=2 studies). All interventions targeted Indigenous cancer survivors and/or community members, with 10 studies (37%) also targeting healthcare providers (see Table 3).

**Table 3:** Interventions aims and participant target groups

Study (Author, year)	Aims				Target groups	
	Support and improve healthcare journey	Increase knowledge	Psycho-social support	Promote dialogue about cancer	Cancer survivors/community members	Health care providers
	(*n*=19)	(*n*=8)	(*n*=6)	(*n*=2)	(*n*=24)	(*n*=10)
Bierbaum (2017)	✓					✓
Burhansstipanov (2012)	✓				✓	✓
Burhansstipanov (2014)	✓				✓	✓
Cuesta-Briand (2015)	✓				✓	✓
Cuesta-Briand (2016)	✓		✓		✓	✓
Cueva (2005)		✓		✓	✓	
Cueva (2010)				✓	✓	
Dignan (2005)	✓				✓	
Dockery (2018)	✓				✓	
Doorenbos (2010)			✓		✓	
Elliott (1999)	✓	✓			✓	✓
Ferris (2015)			✓		✓	
Guadagnolo & Boylan (2011)	✓				✓	
Guadagnolo & Cina (2011)	✓				✓	
Hill (2010)	✓	✓			✓	✓
Hodge (2012)	✓				✓	
Hodge (2016)	✓				✓	
Ivers (2019)	✓				✓	✓
Krebs (2013)	✓	✓			✓	✓
Mokuau (2008)		✓	✓		✓	
Mokuau (2012)		✓	✓		✓	
Petereit (2008)	✓	✓			✓	
Petereit (2011)	✓				✓	
Pruthi (2013)	✓				✓	
Sabesan (2012)	✓				✓	
Sanderson (2010)		✓			✓	✓
Warson (2012)			✓		✓	

### Ingredients of the interventions

We identified seven different ingredients of the interventions: community meetings (*n*=8 studies) [23, 42–44, 47, 49, 54, 66]; patient navigation (*n*=8 studies) [45–48, 52, 56, 65, 67]; visual and performing arts (*n*=7 studies) [55, 58–62, 64]; printed, online, or audio materials (*n*=7 studies) [23, 49, 55, 56, 60, 63, 68]; healthcare provider education (*n*=4 studies) [42, 44, 63, 68]; support groups (*n*=4 studies) [50, 51, 57, 67]; and telehealth (*n*=3 studies) [52, 53, 57]. Studies used one or two of the ingredients with just over half (*n*=14) using two ingredients. For example, Burhansstipanov et al. held community meetings and healthcare provider education [42], whereas Hodge et al. used visual art and printed materials [55, 60]. Visual arts included videos [55, 58, 60] and performing arts involved *Readers Theatre* where cancer survivors read out loud plays that were scripted with cancer stories [59, 62]. Healthcare provider education involved didactic and interactive face-to-face seminars, practice, and online webinars [42, 44, 63, 68].

### Study outcomes

Overall, 89% (*n*=24) of studies showed positive influences of the interventions on outcomes. For example Dignan et al. reported that the proportion of Indigenous women that had a mammogram for breast cancer screening was significantly higher in the intervention group of a randomized control trial in the USA [56]. Results of the pre/post-survey studies that evaluated the *Native Navigators and the Cancer Continuum (NNACC)* [42–44] intervention reported increases in cancer screening, appointments, referrals rates, and knowledge about cancer and cancer care. Two studies reported no impact from the intervention [61, 65], and one study reported negative findings in the form of unresolved tensions between Western and Indigenous people in delivering of the intervention [50].

Twenty-two (81%) studies evaluated outcomes that aligned *holistic wellness* (physical, mental, emotional, and spiritual), and 12 studies (44%) evaluated outcomes related to *responses towards the intervention*. Both *holistic wellness* and *responses towards the intervention* were evaluated in seven studies [42, 44, 49, 60, 63, 64, 67], but only five studies evaluated *responses towards the intervention* [50, 52, 57, 61, 68]. (See Table 4 for the intervention ingredients with study outcomes and Table 5 for descriptions of outcomes).

**Table 4:**
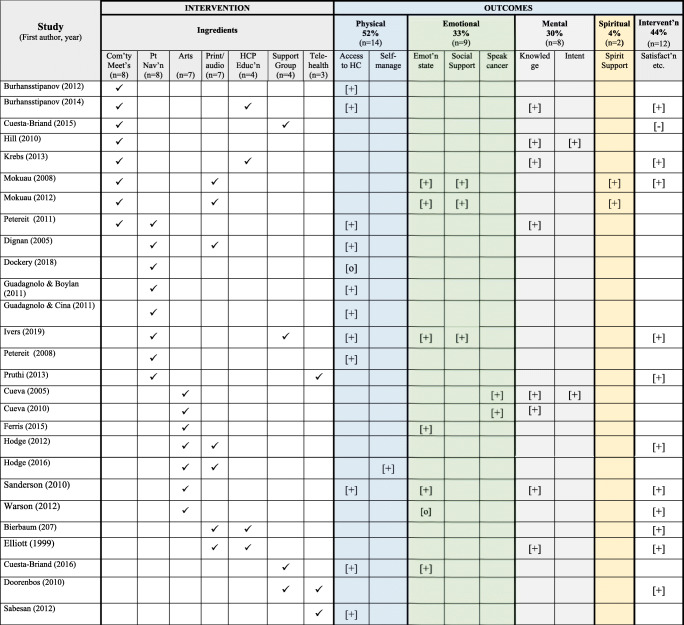
Intervention ingredients and study outcomes * [+] positive influence; [o] no influence or inconclusive; [−] negative influence

**Table 5:** Descriptions of outcomes (*n*=27 studies)

Study	Descriptions of outcomes (Wellness Outcomes Categories*)
Bierbaum (2017)	•Majority of participants agreed or strongly agreed that the flipchart and flyer were valuable, culturally appropriate, useful for explaining cancer and the Aboriginal cancer patient journey *(R)*
Burhansstipanov (2012)	•Increased scheduling and attending cancer screening/diagnostic appointments *(P)*
Burhansstipanov (2014)	•Improved referrals and access to care *(P)* •Increased knowledge by 28% *(M)* •Workshop content identified as useful by 90% of participants; 92.3% would recommend to others *(R)*
Cuesta-Briand (2015)	•Unresolved tensions identified between mainstream and Indigenous people for delivering the program included: (1) flexibility and resistance to formal structuring, (2) understanding of confidentiality *(R)*
Cuesta-Briand (2016)	•Increased access to cancer services *(P)* •Fostered social interaction and built relationships *(E)*
Cueva (2005)	•66.1% shared they learned about cancer (K) •61.7% intended to change their behavior (K) •94.5% felt more comfortable talking about cancer *(E)*
Cueva (2010)	•Improved knowledge, attitudes, beliefs (K) •Improved engagement in meaningful conversations *(E)*
Dignan (2005)	•Increase in the proportion of women having a mammogram within 12 months (*p*=0.013) *(P)*
Dockery (2018)	•No statistically significant change in initiation or completion of treatment *(P)*
Doorenbos (2010)	•High level of satisfaction with intervention *(R)*
Elliott (1999)	•Increased knowledge (*p* = 0.000) *(M)* •92% agreement the intervention was culturally sensitive *(R)*
Ferris (2015)	•Increased calm (*p* = 0.04); relaxed (*p* = 0.02); content (*p* < 0.01) *(E)*
Guadagnolo & Boylan (2011)	•Decreased # treatment interruption days (mean, 1.7 days; 95% CI, 1.1–2.2 days) *(P)*
Guadagnolo & Cina (2011)	•Improved satisfaction with health care services (*p*<.0001) *(P)*
Hill (2010)	•Increased knowledge (*p*<0.01); improved attitude (*p*<0.05) *(M)* •Very likely/extremely likely to engage in behavioral change to ↓ cancer risk *(M)*
Hodge (2012)	•Favorable views of toolkit materials—perceived to be relevant, informative, and easy to understand *(R)*
Hodge (2016)	•Improved pain management (*p*=.02) *(P)*
Ivers (2019)	•Improved access to cancer care services *(P)* •Helped improve wellbeing *(E)* •Services viewed as being culturally safe *(R)*
Krebs (2013)	•Increased knowledge by 28.4% *(M)* •Workshops perceived as fun, productive, interactive and effective *(R)*
Mokuau (2008)	•Improved self-efficacy and coping *(E)* •Social Support (*p* < .05), Mobilizing Family (*p* =.05) *(E)*•Spiritual Support (*p* < .05) *(S)* •Participants appreciated cultural tailoring of intervention *(R)*
Mokuau (2012)	•Increased proportion of women performing breast self-exams *(P)* •Improvements in: Self Efficacy (*p* = .001), Coping (*p* = .05), *(E)* •Social Support (*p* < .001), Mobilizing Family (*p* =.002) *(E)* •Spiritual Support (*p* = .002) *(S)*
Petereit (2011)	•Decreased # treatment interruption days (mean, 1.7 days; 95CI, 1.1–2.2 days) *(P)*•Increased knowledge levels in cervical cancer (*p* < 0.001), breast cancer (*p*<0.001), prostate cancer (*p*<0.001), and colorectal cancer (*p* < 0.001) *(M)*
Petereit (2008)	•Decreased treatment interruptions (*p* = .002) *(P)*
Pruthi (2013)	•Patient satisfaction good or excellent by 98% of participants *(R)*
Sabeson (2012)	•Increased specialist consultations and care utilized in hometowns *(P)*
Sanderson (2010)	•Improved selection and adherence to treatment regimen *(P)*•Improved understanding of treatment choices and asking questions *(M)* •Reduced anxiety about treatment *(E)* •Cultural images and graphics in video were culturally relevant *(R)*
Warson (2012)	•Survey determined to be culturally biased and inconclusive *(E)* •Reinforced a native concept of wellness that focused on the complex interaction between mind, body, spirit, and context *(R)*

*Wellness outcomes categories: *P* physical, *M* mental, *E* emotional, *S* spiritual, *R* response to intervention

### Holistic wellness outcomes

The majority of holistic wellness outcomes that were evaluated related to physical aspects of wellness (*n*=14; 64%) [23, 42, 43, 45–48, 51, 53, 55, 64, 65, 67, 69], such as access to healthcare services, self-management of pain, or breast self-exams. Nine studies evaluated outcomes aligned with emotional wellness, such as self-efficacy and coping [23, 49], emotional and social support [23, 49, 67], calmness [58], anxiety [64], stress [61], and comfort speaking about cancer [59, 62]. Eight studies (38%) evaluated mental wellness that involved knowledge [42, 44, 47, 54, 59, 62–64] or intention [54, 62]. Two studies evaluated spiritual wellness and showed a positive impact [23, 49].

No studies evaluated all four dimensions of holistic wellness, though three studies [23, 49, 64] evaluated three dimensions of wellness. For example, Mokuau et al. evaluated physical, emotional, and spiritual outcomes with Native Hawaiian women [23, 49] and Sanderson et al. evaluated physical, mental, and emotional outcomes with Navajo women [64]. Half the studies (*n*=12) assessed only one dimension of wellness [43–46, 48, 53–56, 58, 63–65], whereas six studies [42, 47, 51, 59, 62, 67] measured two aspects of wellness.

### Responses towards the intervention that influence wellness

Of the 12 studies that evaluated a response towards the intervention, only one reported a negative response [50]. Participants in Cuesta-Briand et al.’s qualitative study in Australia identified unresolved tensions between Indigenous and non-Indigenous people around the structure and delivery of the intervention, which threatened the intervention’s success and sustainability [50]. Positive responses towards the interventions included patient satisfaction, perceptions of cultural safety, appropriateness, usefulness, and acceptability of the interventions [42, 44, 52, 57, 60]. For example, Burhansstipanov [42] reported workshop content was perceived as useful by 90% of participants and Pruthi [52] identified patient satisfaction as good or excellent by 98% of participants.

Aspects of cultural relevance or safety were explicitly reported in six studies [49, 61, 63, 64, 66, 68]. Participants in the study by Bierbaum et al. [68] considered intervention resources to be culturally appropriate and acceptable and Mokuau et al.’s [49] participants appreciated cultural tailoring of materials. Although participants in the pilot study by Warson et al. [61] described the intervention positively, the survey used for data collection was considered culturally biased and therefore inconclusive.

## Discussion

The purpose of this systematic review was to synthesize the published research evidence on cancer survivorship interventions that have been conducted with Indigenous Peoples. In total, we found 20 different interventions that were evaluated in 27 published studies. The majority of studies (89%) showed a positive impact on the outcomes evaluated. Most (81%) were published in the last 10 years with the majority (77%) in the USA. Participants represented a diversity of Indigenous Peoples and were predominately female. Doorenbos et al. [57] suggest that the lack of male participation in cancer survivorship interventions may be related to cultural differences in self-expression or discomfort in mixed-gender groups. Given that males may experience survivorship differently, and that sex and gender have historically been foundational to roles, traditions, and ceremonies for many Indigenous Peoples, further understandings of sex and gender conceptualizations and expressions is warranted in designing and delivering cancer survivorship interventions. We recommend further research to understand sex and gender conceptualizations and expressions in designing and delivering cancer survivorship interventions for Indigenous Peoples.

### Relevance of the interventions to Indigenous communities

Studies in this review did not provide strong evidence that the interventions had relevance to the Indigenous communities that participated in them, with just over half (60%) rating as moderately relevant, and one study [53] proving no evidence of community relevance. Ratings of relevance were based on the published papers providing sufficient details that the study was developed collaboratively with Indigenous communities. However, it is unclear whether authors had been engaged but failed to report their engagement in sufficient details to warrant high ratings. Smylie et al. 2016 [33] noted similar limitations on the role of Indigenous community’s participation in prenatal and infant-toddler programs, noting that community investment, cultural integrity, and relevance were unclear because of inadequate reporting of details and context. From a social justice standpoint, Indigenous research requires methodologies that engage with communities and give back in ways that community members decide what is “useful” and relevant [71]. We support recommendations from Indigenous scholars that reporting how Indigenous communities and/or participants are involved in a study and the relevance to communities be a priority in publishing [32, 33]. As of December 2020, the *Canadian Journal of Public Health* requires authors to clearly describe how Indigenous Peoples were engaged in a study to be considered for publication, becoming the first scientific journal known in Canada to adopt such a policy [72].

The studies that evaluated the *Walking Forward* - *To’katakiya zanniyan omani pi ye/yo* intervention [45–48] offered ways of achieving community relevance by describing their engagement methods with participating Hawaiian communities. These included participation in the planning process, implementation, consultations, and shared responsibilities for data analyses, writing, and dissemination of findings. Similar methods and strategies were described in a *“Two-Eyed Seeing”* approach by Rowan et al. [41] of cultural interventions to treat addictions with Indigenous populations. Developing Indigenous health research requires Western researchers to create shared spaces that legitimize Indigenous knowledge, acknowledge the tainted history of research with Indigenous Peoples, and recognize the inherent rights of Indigenous Peoples to self-determine knowledge for understanding the world [71].

Researchers wanting to be engaged in ethical research with Indigenous communities can look for guidance in policies and principles that have been established. For example, in Canada, the Tri-Council policy statement *Research Involving the First Nations, Inuit, and Metis People of Canada* emphasizes traditional cultural values, community engagement, and mutually respectful relationships. The Public Health Agency of Canada further suggests the following for developing interventions with Indigenous communities: (1) be based in community, (2) use a holistic approach, (3) integrate Indigenous cultural knowledge, (4) build on community strengths and needs, (5) develop partnerships/collaboration, and (6) demonstrate effectiveness [73]. These strategies are consistent with the community relevance assessments we used in this review and in studies by Minichiello et al. [32] and Smylie et al. [33]. Establishing an equitable research environment is necessary to guide meaningful cancer survivorship research with Indigenous communities.

### Methodological approaches

Studies in this review commonly used quasi-experimental designs and many involved data collection methods, such as surveys, that do not typically represent Indigenous epistemologies or approaches to knowledge development [71]. Warson reported that American Indian and Alaska Native participants of an art intervention responded positively to the intervention but were not receptive to completing the validated survey as it was culturally biased [61]. Valuable insights can be gleaned from understanding the inherent tensions between Western science and Indigenous approaches to knowledge development [71]. Many Western approaches uphold neutrality, objectivity, and universal laws of generalizability, concepts that may philosophically conflict with Indigenous research paradigms. These conflicts were underscored in the study by Cuesta-Briand et al., who reported opposing perspectives on the structure and delivery of the intervention as central to the tensions between Indigenous and Western researchers and participants [66].

Studies in this review used a variety of data collection methods that are consistent with Indigenous approaches to knowledge sharing, such as interviews [51, 59, 63, 64, 66, 67], discussion groups [60, 62, 63], and art [61]. However, theoretical perspectives that give ownership to Indigenous communities and do not separate the research from their ways of knowing were not explicit. Instead, studies predominately described Western research methodologies. Kovach explains that researchers wishing to use Indigenous methodologies alongside Western approaches should transparently indicate this, highlight differences, and not assume that Indigenous methodologies can be subsumed under Western ways of knowing [71].

### Outcomes and positioning to holistic wellness

While the inclusion of body, mind, emotions, and spirit is widely recognized as integral to wellness among Indigenous Peoples [24, 74], studies in this review were predominately focused on the physical aspect of wellness, which is more consistent with Western biomedical healthcare perspectives. Frameworks such as the *First Nations Mental Wellness Continuum* [26] and *Wellbeing Framework for Australian Aboriginal and Torres Strait Islander Peoples* [75] offer conceptualizations of Indigenous wellness that can guide meaningful measurement approaches and outcomes. Indigenous-led research and decolonizing approaches that include Elders, healers, Knowledge Keepers, and community members will help to regain access to knowledge for holistic health and healing [71, 74].

### Responses about the intervention

**Fig. 2:**
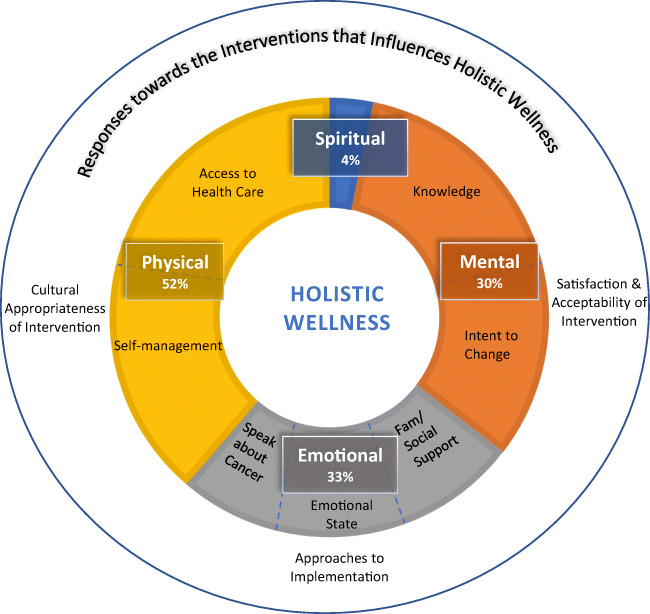
Holistic wellness and response to the intervention outcomes identified in the literature

Despite relatively low ratings for relevance to Indigenous communities, participants predominately described the interventions as acceptable and culturally sensitive. These findings indicate that the interventions themselves were well-received, with many participants stating they would recommend the interventions to other community members. We summarized responses to the interventions as impacting wellness through participants’ engagement with the interventions. For example, although the survey in Warson’s study was determined to be culturally biased, the art intervention focused on a native concept of wellness and was positively received [61], while Sanderson et al. reported Navajo women found the video intervention to be culturally specific and positive [64]. These findings illustrate the importance of engaging with Indigenous communities to develop, deliver, and evaluate cancer survivorship interventions. Figure 2 graphically summarizes the holistic wellness outcomes and response to the intervention identified in the literature.

### Strengths and limitations of the review

As a team of Western researchers and Indigenous community members, we followed a systematic and rigorous process with regular community meetings to conduct this review and we privileged Indigenous knowledge in the analysis and interpretations of findings. Limitations, however, must be acknowledged. While reference lists of included studies were examined for further studies, we did not search gray literature, so findings were restricted to scholarly journals. We did not contact primary authors to clarify study relevance to Indigenous communities, and therefore our ratings may be low due to underreporting in the published articles. Lastly, we did not exclude studies based on methodological weaknesses or ratings of indigenous relevance, nor did we analyze data for different Indigenous groups. Rather, we synthesized all studies together to provide a summary of the research to date.

## Conclusions

Indigenous Peoples have shown resilience in their adaptations to the traumas of colonization that have contributed to lack of culturally safe cancer survivorship care. We found few studies, and the studies we found only represented a small number of Indigenous Peoples. Methodological quality in these studies was generally low, based on Western standards, and more importantly, they did not meet relevancy standards in reporting of engagement with communities. To improve the cancer survivorship journey for Indigenous people, we need research that is relevant to Indigenous communities, culturally safe and effective, and honoring the diverse conceptualizations of health and wellness among Indigenous Peoples.

## Supplementary Information

ESM 1 (DOCX 54 kb)

ESM 2 (DOCX 27 kb)

## Data Availability

Additional File 1: Search strategy; Additional File 2: Search Filters

## Footnotes

1 Consistent with international health research environments, the term “Indigenous” is used throughout this paper to denote the original habitant of a country regardless of its borders. We refer to specific Indigenous Peoples when identifiable, and use the term Indigenous when a group is not identifiable or when referring to literature about the original inhabitants of a country.
