# Supplementary material for: Interventions to improve cancer survivorship among Indigenous Peoples and communities: a systematic review with a narrative synthesis
Source: Support Care Cancer. 2021 May 24;29(11):7029–48. doi: 10.1007/s00520-021-06216-7 (PMC8464576; doi:10.1007/s00520-021-06216-7)
Supplement: Supplementary file 2 — (DOCX 27 kb) [file 520_2021_6216_MOESM2_ESM.docx]

**Additional File 2**

**Filters (references)**

1. Medline

Campbell, Sandy, Marlene Dorgan and Lisa Tjosvold. Filter to Retrieve Studies Related to Indigenous People of Canada the OVID Medline Database. John W. Scott Health Sciences Library, University of Alberta. Rev. March 8, 2016. <http://guides.library.ualberta.ca/ld.php?content_id=14026803>

Campbell, Sandy, Marlene Dorgan and Lisa Tjosvold. Filter to Retrieve Studies Related to Indigenous People of Canada’s North (including Northern Quebec and Labrador) OVID MEDLINE Database. John W. Scott Health Sciences Library, University of Alberta. Rev. February 8, 2016. <http://guides.library.ualberta.ca/ld.php?content_id=13959040>

Campbell, Sandy, Marlene Dorgan and Lisa Tjosvold. Filter to Retrieve Studies Related to Indigenous People of Alberta in the OVID Medline Database. John W. Scott Health Sciences Library, University of Alberta. Rev. December 19, 2013. Updated. February 8, 2016. <http://guides.library.ualberta.ca/ld.php?content_id=14026775>

Campbell, Sandy, Marlene Dorgan and Lisa Tjosvold. Filter to Retrieve Studies Related to Indigenous People of British Columbia the OVID Medline Database. John W. Scott Health Sciences Library, University of Alberta. Rev. February 25, 2017. <http://guides.library.ualberta.ca/ld.php?content_id=14026803>

Campbell, Sandy, Marlene Dorgan and Lisa Tjosvold. Filter to Retrieve Studies Related Indigenous Peoples of Manitoba in the OVID MEDLINE Database. John W. Scott Health Sciences Library, University of Alberta. Rev. May 22, 2013. <http://guides.library.ualberta.ca/aecontent.php?pid=448005>

Campbell, Sandy, Marlene Dorgan and Lisa Tjosvold. Filter to Retrieve Studies Related to Indigenous People of New Brunswick from the OVID MEDLINE Database. John W. Scott Health Sciences Library, University of Alberta. Rev. March 8, 2016 <http://guides.library.ualberta.ca/ld.php?content_id=14026859>

Campbell S, Dorgan M and Tjosvold L. Filter to Retrieve Studies Related to Indigenous People of Newfoundland and Labrador the Ovid Medline Database. John W. Scott Health Sciences Library, University of Alberta. Rev. March 8, 2016. <http://guides.library.ualberta.ca/ld.php?content_id=14026878>

Campbell, Sandy, Marlene Dorgan and Lisa Filter to Retrieve Studies Related to Indigenous People of the Northwest Territories OVID Medline Database. John W. Scott Health Sciences Library, University of Alberta. Rev. Dec 16, 2013. <http://guides.library.ualberta.ca/ld.php?content_id=14027565>

Campbell, Sandy, Marlene Dorgan and Lisa Tjosvold. Filter to Retrieve Studies Related to Indigenous People of Nova Scotia in OVID Medline Database. John W. Scott Health Sciences Library, University of Alberta. Rev. May 22, 2013. <http://guides.library.ualberta.ca/ld.php?content_id=14027030>

Campbell S, Dorgan M and Tjosvold L. Filter to Retrieve Studies Related to Indigenous People of Nunavut in the Ovid MEDLINE Database. John W. Scott Health Sciences Library, University of Alberta. Rev. Dec. 11, 2013 <http://guides.library.ualberta.ca/ld.php?content_id=14027086>

Lisa Tjosvold, Sandy Campbell and Marlene Dorgan. Filter to Retrieve Studies Related Indigenous Peoples of Ontario in the OVID MEDLINE Database. John W. Scott Health Sciences Library, University of Alberta. Rev. Dec. 18, 2013. <http://guides.library.ualberta.ca/ld.php?content_id=14027103>

Lisa Tjosvold, Sandy Campbell and Marlene Dorgan. Filter to Retrieve Studies Related Indigenous Peoples of Quebec in the OVID MEDLINE Database. John W. Scott Health Sciences Library, University of Alberta. Rev. Dec. 20, 2013. <http://guides.library.ualberta.ca/ld.php?content_id=14027124>

Dorgan, Marlene, Lisa Tjosvold and Sandy Campbell. Filter to Retrieve Studies Related Indigenous Peoples of Saskatchewan in the OVID MEDLINE Database. John W. Scott Health Sciences Library, University of Alberta. Rev. December 17, 2013. <http://guides.library.ualberta.ca/ld.php?content_id=14027138>

Campbell, Sandy, Marlene Dorgan and Lisa Tjosvold. Filter to Retrieve Articles Related to Indigenous Peoples of the Yukon Territory from the OVID MEDLINE Database. John W. Scott Health Sciences Library, University of Alberta. Rev. Dec. 20, 2013. <http://guides.library.ualberta.ca/ld.php?content_id=14027164>

1. Embase

Campbell, Sandy, Marlene Dorgan and Lisa Tjosvold. Filter to Retrieve Studies Related to Indigenous People of Alberta in the OVID EMBASE Database. John W. Scott Health Sciences Library, University of Alberta. Rev. March 14, 2016. <http://guides.library.ualberta.ca/ld.php?content_id=14026775>

Campbell, Sandy, Marlene Dorgan and Lisa Tjosvold. Filter to Retrieve Studies Related to Indigenous People of Canada the OVID EMBASE Database. John W. Scott Health Sciences Library, University of Alberta. Rev. March 14, 2016. <http://guides.library.ualberta.ca/ld.php?content_id=14026803>

Campbell, Sandy, Marlene Dorgan and Lisa Tjosvold. Filter to Retrieve Studies Related to Indigenous People of Alberta in the OVID EMBASE Database. John W. Scott Health Sciences Library, University of Alberta. Rev. March 14, 2016. <http://guides.library.ualberta.ca/ld.php?content_id=14026775>

Campbell, Sandy, Marlene Dorgan and Lisa Tjosvold. Filter to Retrieve Studies Related Indigenous Peoples of Manitoba in the OVID MEDLINE Database. John W. Scott Health Sciences Library, University of Alberta. Rev. March 14, 2016. <http://guides.library.ualberta.ca/ld.php?content_id=14026847>

Campbell, Sandy, Marlene Dorgan and Lisa Tjosvold. Filter to Retrieve Studies Related to Indigenous People of New Brunswick from the OVID EMBASE Database. John W. Scott Health Sciences Library, University of Alberta. Rev. March 14, 2016 <http://guides.library.ualberta.ca/ld.php?content_id=14026859>

Campbell S, Dorgan M and Tjosvold L. Filter to Retrieve Studies Related to Indigenous People of Newfoundland and Labrador the Ovid Medline Database. John W. Scott Health Sciences Library, University of Alberta. Rev. March 8, 2016. <http://guides.library.ualberta.ca/ld.php?content_id=14026878>

Dorgan, Marlene, Lisa Tjosvold and Sandy Campbell. Filter to Retrieve Studies Related Indigenous Peoples of Saskatchewan in the OVID MEDLINE Database. John W. Scott Health Sciences Library, University of Alberta. Rev. December 17, 2013. <http://guides.library.ualberta.ca/ld.php?content_id=14027138>
